# Supplementary material for: Multiple Dosing and Preactivation of Mesenchymal Stromal Cells Enhance Efficacy in Established Pneumonia Induced by Antimicrobial-Resistant Klebsiella pneumoniae in Rodents
Source: Int J Mol Sci. 2023 Apr 29;24(9):8055. doi: 10.3390/ijms24098055 (PMC10179238; doi:10.3390/ijms24098055)
Supplement: Supplementary file 1 [file ijms-24-08055-s001.zip › ijms-2223057-supplementary.pdf]

Supplementary Figure S1:

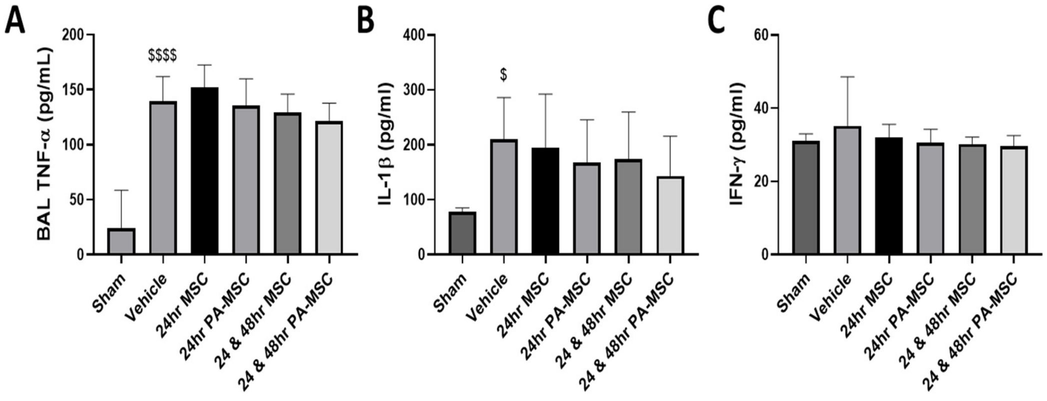

Supplementary Figure S1. ELISAs on BAL inflammatory cytokines TNF-α, IL-1β, and IFN-γ showed that they were unchanged between treatment groups (A – C). TNF = Tumour Necrosis Factor; IL = Interleukin; IFN = Interferon. Columns represent mean. Error bars represent SD. $, $$$$ = $p \leq 0.05$, 0.0001 versus sham group. n = 8-12.

Supplementary Figure S2:

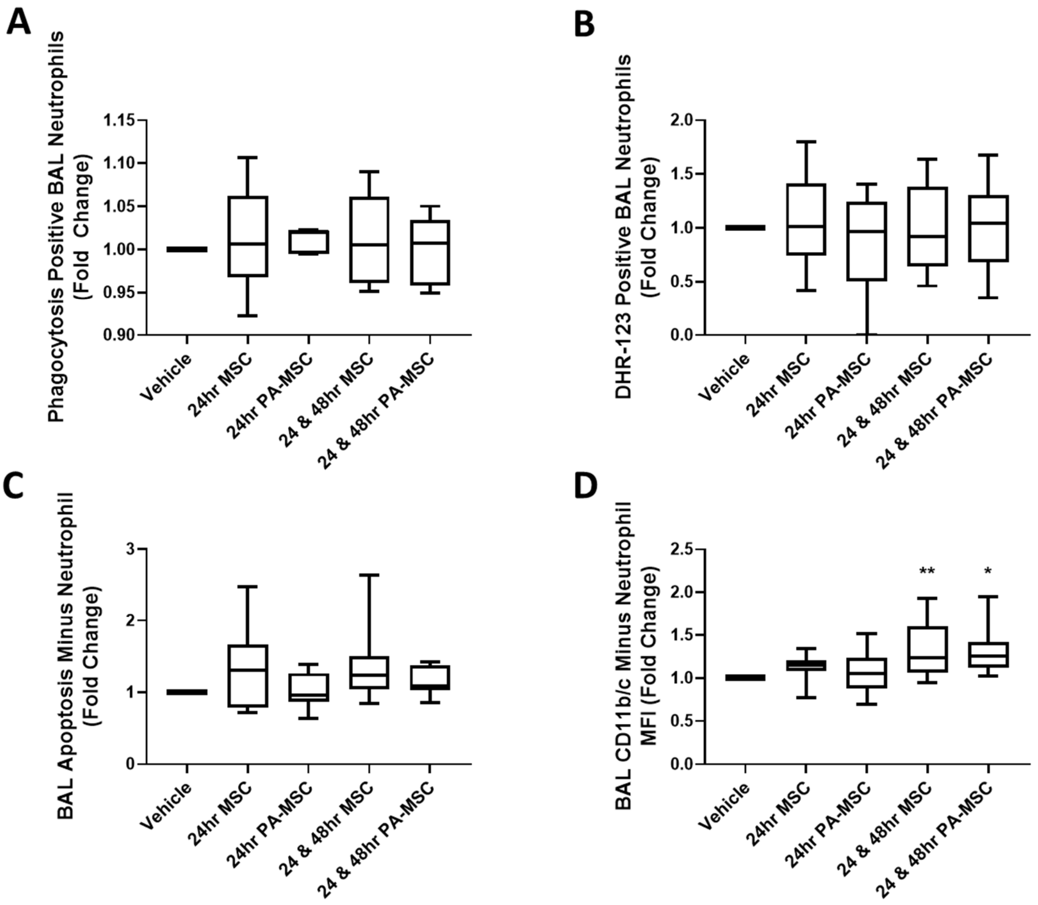

Supplementary Figure S2. BAL neutrophil phagocytosis (A) and superoxide anion production (B) was unchanged between treatment groups, as analysed by flow cytometry. The other cells in the BAL, minus neutrophils, showed no change in the rate of apoptosis (C) but did have a significantly increased expression of CD11b/c (D). BAL = bronchoalveolar lavage. Box plots and whiskers represent minimum, first quartile, median, third quartile, and maximum. *, **, = $p \leq 0.05$, 0.01, versus vehicle control. n = 8-12.

Supplementary Figure S3:

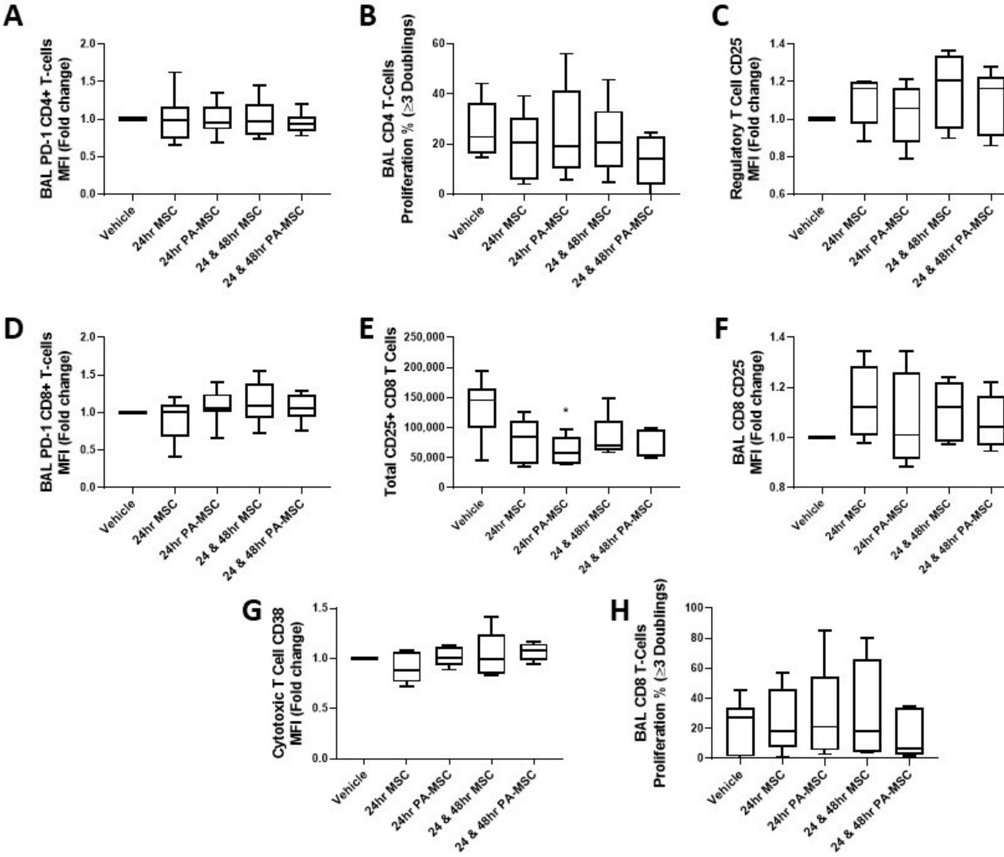

Supplementary Figure S3. Flow cytometry analysis showed that there was no significant change in either the expression level of PD-1 on CD4+ T helper cells (A), the proliferation rate (B), or on the expression level of CD25 on regulatory T-cells (C) between treatment groups in the BAL. BAL CD8+ cytotoxic T-cells had no change in the expression level of PD-1 between treatment groups (D), with only the single dose of cytomix licensed MSCs (PA-MSCs) significantly reducing the total number of PD-1 positive cytotoxic T-cells (E). There was no change in either the expression level of CD25 on CD8+ memory T-cells (F), the expression level of CD38 on cytotoxic T-cells in the BAL (G), or on the proliferation rate (H). BAL = bronchoalveolar lavage. Box plots and whiskers represent minimum, first quartile, median, third quartile, and maximum. *, = $p \leq 0.05$, versus vehicle control. n = 8-12.

Supplementary Figure S4:

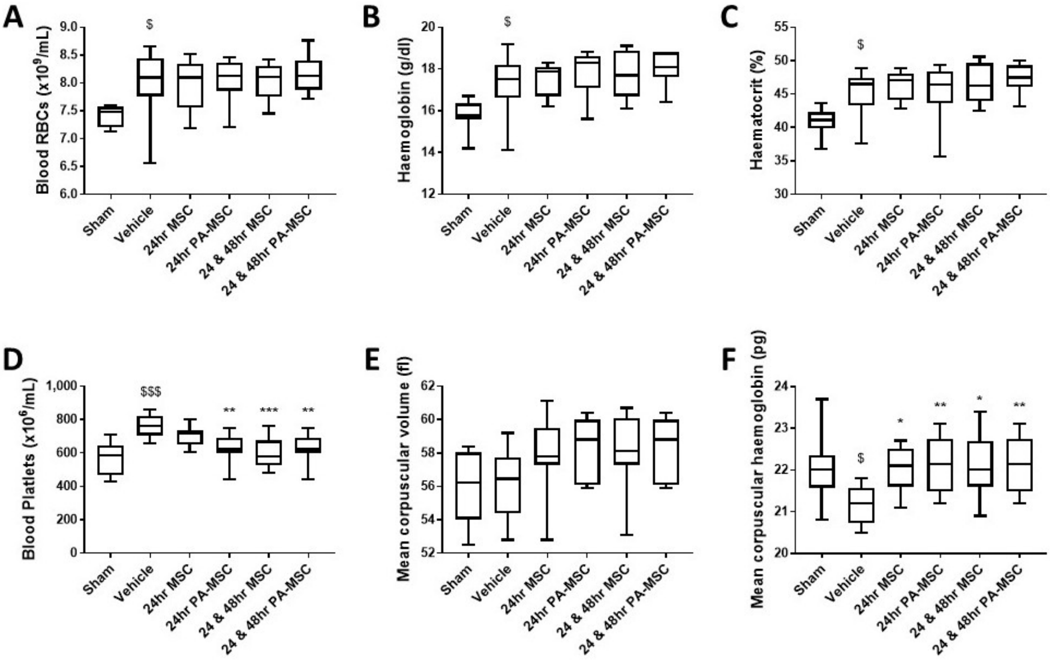

Supplementary Figure S4. Haemoanalyser showed that there was a significant injury affecting haematological parameters, Total RBC (A), Haemoglobin (B), and Haematocrit (C) when comparing vehicle to sham, but there was no change between treatment groups. Blood platelets were returned to sham levels after PA-MSC and repeated doses of naive MSC (MSC) administration (D). Mean corpuscular volume was unchanged across treatment groups and sham (E). Mean corpuscular haemoglobin was significantly reduced between vehicle and sham (F) with all treatment groups significantly elevating it, returning to sham levels. Box plots and whiskers represent minimum, first quartile, median, third quartile, and maximum. *, **, ***, = $p \leq 0.05$, 0.01, 0.001 versus vehicle control. $, $$$ = $p \leq 0.05$, 0.001 versus sham group. n = 8-12.

Supplementary Figure S5:

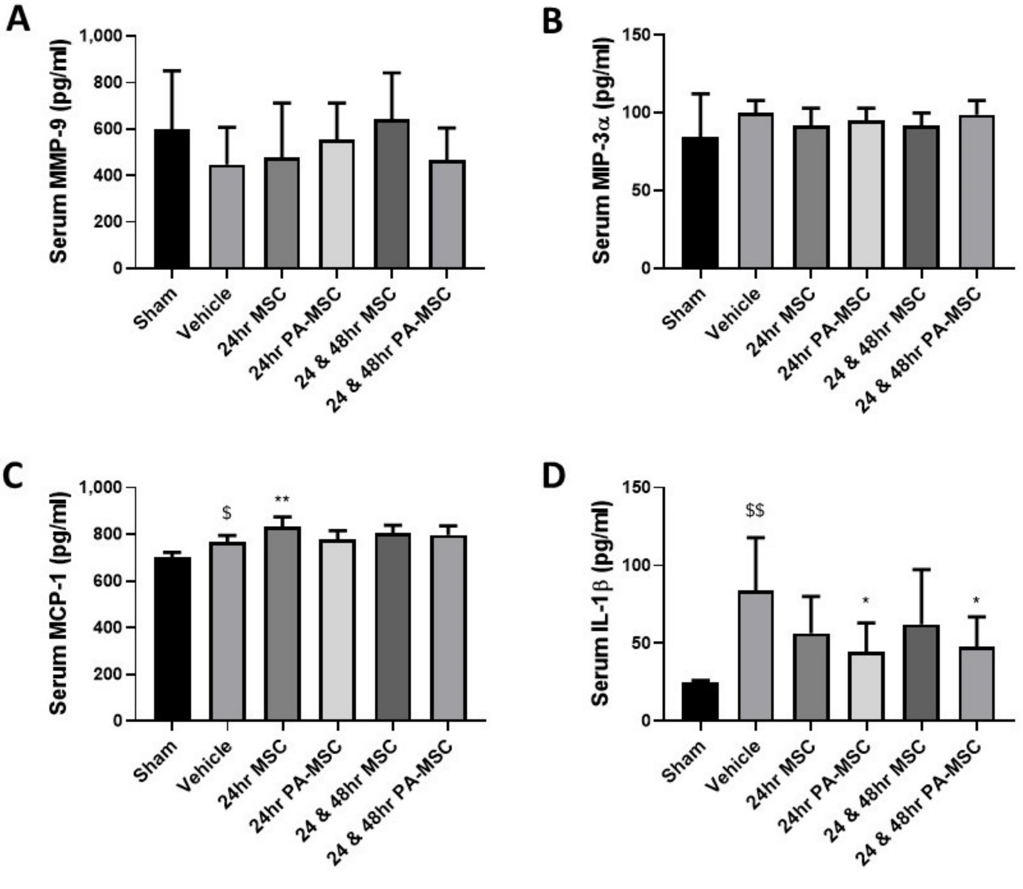

Supplementary Figure S5. ELISAs on serum inflammatory cytokines MMP-9 and MIP-3α showed that they were unchanged between treatment groups (A & B). MCP-1 was significantly elevated in the single dose MSC group compared to vehicle control (C). Inflammatory cytokine IL-1β was significantly increased in vehicle compared to sham with either PA-MSC dose significantly reducing its level in the serum (D). MMP = Matrix metalloprotease; MIP = Macrophage inflammatory protein; MCP = Monocyte chemotactic protein; IL = Interleukin. Columns represent mean. Error bars represent SD. *, **, = $p \leq 0.05$, 0.01versus vehicle control. $, $$ = $p \leq$ 0.05, 0.01 versus sham group. N = 8-12.

Supplementary Figure S6:

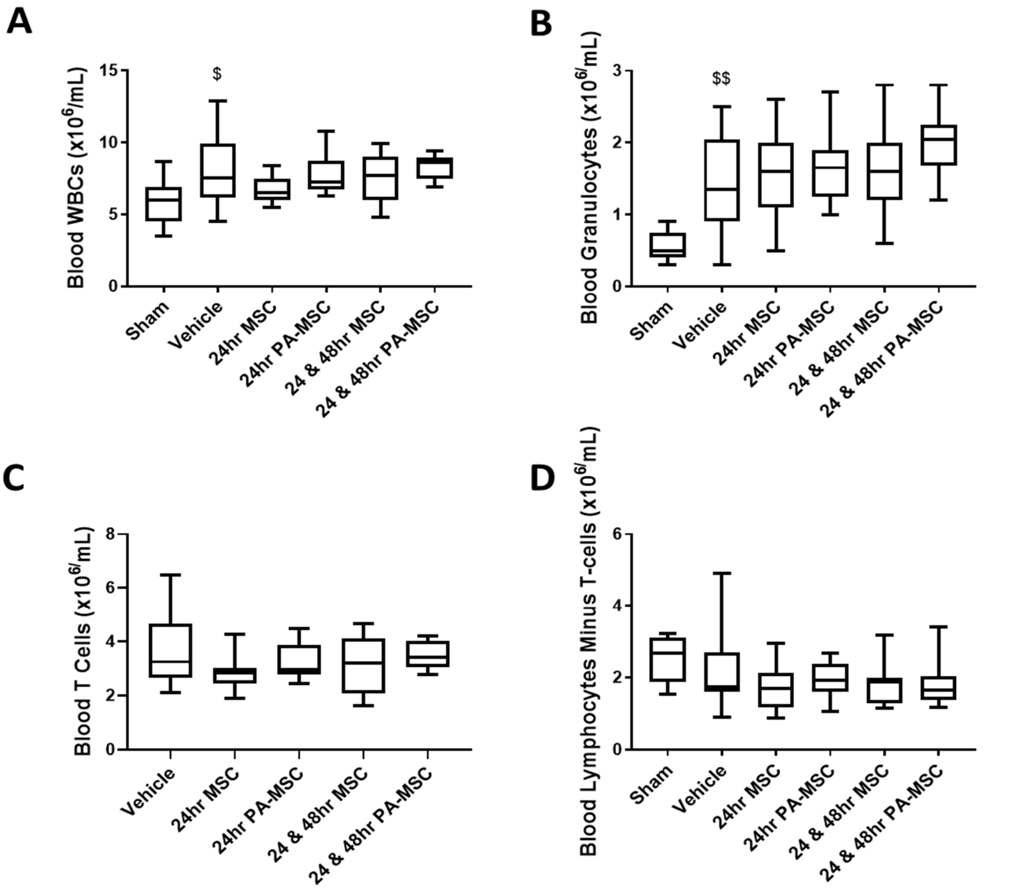

Supplementary Figure S6. There was no change between treatment groups for the total amount of WBCs (A), Granulocytes (B), T-cells (C), and Lymphocytes minus T-cells (D) in the blood. The injury caused an increased number of Granulocytes in the blood when comparing vehicle to sham. Box plots and whiskers represent minimum, first quartile, median, third quartile, and maximum. $, $$, = $p \leq 0.05$, 0.01 versus sham group. n = 8-12.

Supplementary Figure S7:

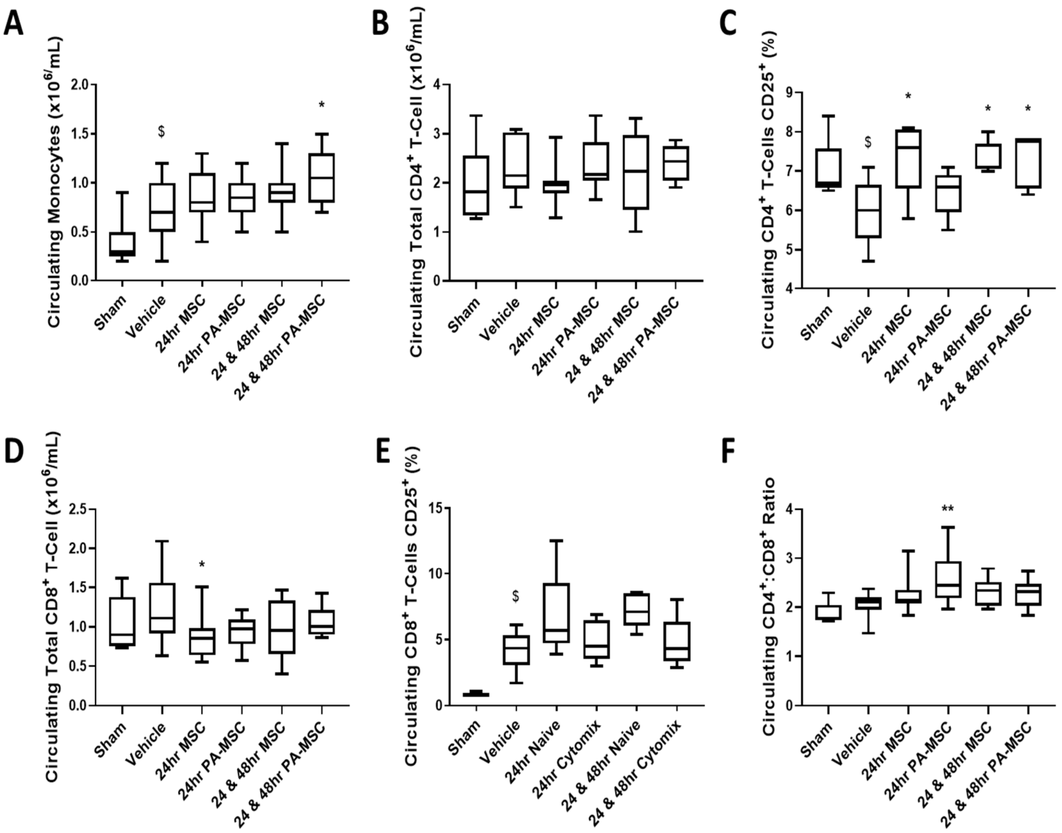

Supplementary Figure S7. The numbers of circulating monocytes was significantly elevated in the vehicle control group compared to sham (A) with repeated doses of PA-MSC treatment significantly increasing it compared to vehicle. Circulating CD4+ T helper cells was unchanged across all treatment groups (B). The proportion of CD4+ regulatory T-cells was returned to healthy control levels after MSC and repeated PA-MSC administration (C). Circulating cytotoxic CD8+ T-cells was significantly reduced after a single MSC dose (D). There was an increased percentage of CD8+ T-cells co-expressing CD25 after bacterial infection compared to shams however, this was unchanged between vehicle and treatment groups (E). The ratio of circulating CD4:CD8 T-cells was significantly increased after a single PA-MSC dose (F). Box plots and whiskers represent minimum, first quartile, median, third quartile, and maximum. *, **, = $p \leq 0.05$, 0.01 versus vehicle control. $ = $p \leq 0.05$ versus sham group. n = 8-12.

Supplementary Figure S8:

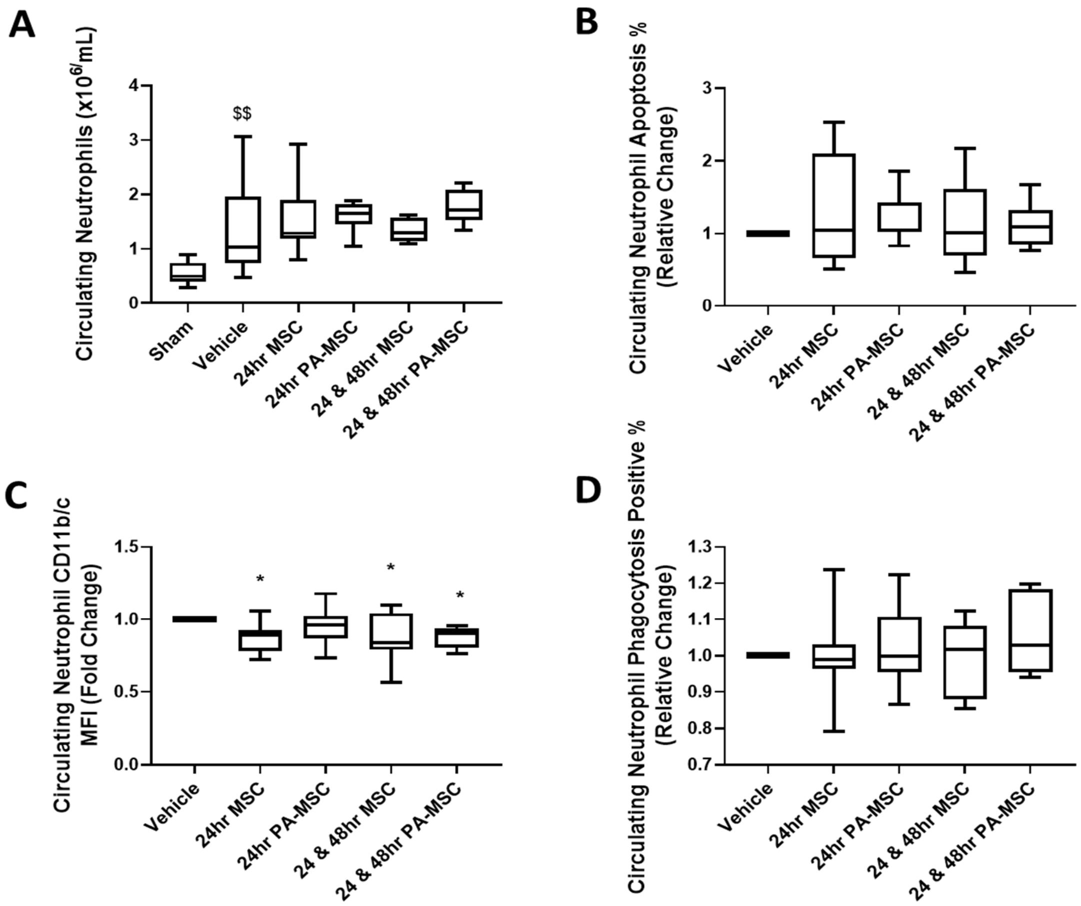

Supplementary Figure S8. Circulating neutrophils were elevated in the injury with no change in the total number (A) or their rate of apoptosis (B) across all treatment groups. The activation state of circulating neutrophils was significantly reduced after either MSC dose and repeated doses of PA-MSC administration (C). Neutrophil phagocytosis was unchanged across all treatment groups (D). Box plots and whiskers represent minimum, first quartile, median, third quartile, and maximum. *= $p \leq 0.05$ versus vehicle control. $$ = $p \leq$, 0.01 versus sham group. n = 8-12.
